# Deep learning for predicting fibrotic progression risk in diabetic individuals with metabolic dysfunction-associated steatotic liver disease initially free of hepatic fibrosis

**DOI:** 10.1016/j.heliyon.2024.e34150

**Published:** 2024-07-05

**Authors:** Ruihong Dai, Miaomiao Sun, Mei Lu, Lanhua Deng

**Affiliations:** Department of Ultrasound, Meng Cheng County Hospital of Chinese Medicine, Bozhou City, Anhui Province, China

**Keywords:** Nonalcoholic fatty liver disease, Type 2 diabetes mellitus, Deep learning, Shear wave elastography, Liver stiffness measurement, Fibrotic progression

## Abstract

**Objective:**

Metabolic dysfunction-associated steatotic liver disease (MASLD) significantly impacts patients with type 2 diabetes mellitus (T2DM), where current non-invasive assessment methods show limited predictive power for future fibrotic progression. This study aims to develop an enhanced deep learning (DL) model that integrates ultrasound elastography images with clinical data, refining the prediction of fibrotic progression in T2DM patients with MASLD who initially exhibit no signs of hepatic fibrosis.

**Methods:**

We enrolled 946 diabetic MASLD patients without advanced fibrosis, confirmed by initial liver stiffness measurements (LSM) below 6.5 kPa. Patients were divided into a training dataset of 671 and a testing dataset of 275. Hepatic shear wave elastography (SWE) images measured liver stiffness, classifying participants based on progression. A DL integrated model (DI-model) combining SWE images and clinical data was trained and its predictive performance compared with individual Image and Tabular models, as well as a logistic regression model on the testing dataset.

**Results:**

Fibrotic progression was observed in 18.1 % of patients over three years. During the training phase, the DI-model outperformed other models, achieving the lowest validation loss of 0.161 and highest accuracy of 0.933 through cross-validation. In the testing phase, it demonstrated robust discrimination with AUCs of 0.884 and 0.903 for the receiver operating characteristic and precision-recall curves, respectively, clearly outperforming other models. Shapley analysis identified BMI, LSM, and glycated hemoglobin as critical predictors.

**Conclusion:**

The DI-model significantly enhances the prediction of future fibrotic progression in diabetic MASLD patients, demonstrating the benefit of combining clinical and imaging data for early diagnosis and intervention.

## 1 Introduction

Metabolic dysfunction-associated steatotic liver disease (MASLD) [1], the latest term for steatotic liver disease associated with metabolic syndrome [2], previously referred to as non-alcoholic fatty liver disease (NAFLD) [3], encompasses a spectrum of liver conditions. These conditions range from mere hepatic steatosis to the more severe metabolic dysfunction-associated steatohepatitis (MASH) [4], representing a critical challenge in global health. At present, MASLD is estimated to affect approximately one quarter of the global adult population, with projections suggesting an increase in the prevalence of NASH from 20 % in 2015 to an anticipated 27 % by 2030 [5]. The progression from NASH to cirrhosis, and potentially to hepatocellular carcinoma, is observed to occur at an accelerated pace among individuals diagnosed with type 2 diabetes mellitus (T2DM) co-existing with MASLD [6]. This particular cohort, comprising about 55 % of the T2DM population, is significantly at increased risk for the advancement of fibrosis, with nearly 17 % presenting advanced stages of fibrosis at initial diagnosis [7]. The coexistence of T2DM and MASLD not only accelerates the progression of fibrosis but also increases the likelihood of progression even without initial fibrotic changes, as evidenced by a longitudinal study revealing a 27 % fibrotic progression rate within three years [8]. Given the high incidence and rapid progression of fibrosis among these patients, precise identification of individuals at increased risk for fibrotic progression is critical for effective clinical management and intervention.

While liver biopsy stands as the gold standard for fibrosis evaluation, its invasiveness restricts regular application in tracking fibrotic progression [9]. Consequently, non-invasive methods, particularly ultrasound-based imaging techniques that are safe and widely available, have become preferred alternatives. Hepatic elastography, noted for its precise accuracy, is now a favored option for the initial assessment of MASLD patients [10]. Liver stiffness measurements (LSM), serving as markers for fibrosis severity, have been proven to effectively exclude advanced fibrosis with a high negative predictive value [11]. However, while LSM can be employed to grade liver fibrosis, its role in predicting fibrotic progression risk among diabetic MASLD patients without initial signs of fibrosis remains limited.

In addressing this challenge, prediction models based on artificial intelligence (AI) technology have emerged as a promising area of exploration. As an important subset of deep learning (DL), convolutional neural networks (CNNs) have demonstrated significant potential for the automated interpretation of medical imagery, utilizing extensive datasets of annotated images for training [12]. Currently, the effectiveness of CNNs in classifying liver fibrosis stages is well-recognized, particularly in the accurate identification of liver cirrhosis (stage F4) [13]. Research has also applied CNNs to exclude subjects without advanced fibrosis (stage < F3), although their performance requires further enhancement [14,15]. Nevertheless, there remains a significant need for further exploration into tracking changes in the risk of fibrotic progression over time.

Considering that CNNs predominantly utilize ultrasound images as a single data source and overlook additional predictive indicators, this study plans to enhance CNN model performance by integrating multiple clinical parameters of patients, which hold promise for predicting dynamic changes in fibrotic progression risk. Consequently, our approach involves integrating ultrasound elastography images of liver parenchyma with patient clinical data within a DL framework. The objective of this study is to rigorously evaluate the efficacy of this combined model. This enhanced model is expected to facilitate the automatic prediction of future fibrotic progression risk in individuals with T2DM and MASLD who do not initially present hepatic fibrosis, thereby facilitating the earlier therapeutic interventions to prevent further liver damage.

## 2 Materials and methods

### 2.1 Study design

This prospective cohort study conformed to the ethical principles outlined in the declaration of Helsinki and secured authorization from the Ethics Committee of Meng Cheng County Hospital of Chinese Medicine (MZY-XJS-201817). All patients provided written informed consent, which included permission for the publication of any images and clinical data in the manuscript. In line with privacy protection standards, the study employed data that was rigorously anonymized, ensuring a comprehensive safeguarding of participant privacy at every stage of the investigation.

### 2.2 Sample size

The sample size for this study was determined using the Power Analysis and Sample Size (PASS) software. With an assumed fibrotic progression incidence rate of 20 %, a significance level (α) of 0.05, and a statistical power (1-β) of 0.90. Given these parameters, the calculated minimum sample size was 175 participants.

### 2.3 Participant recruitment

From March 2018 to December 2020, a total of 1200 adults diagnosed with T2DM and exhibiting signs of MASLD were recruited. Confirmation of MASLD was based on the absence of viral or autoimmune hepatitis and exclusion of secondary causes of hepatic steatosis, such as alcohol intake exceeding 30 g per day for men and 20 g per day for women, or the use of steatogenic medications [16]. Inclusion criteria included an initial LSM value under 6.5 kPa to rule out advanced fibrosis [11], and a commitment to a minimum follow-up period of three years. Individuals were excluded if they had any malignancy, reported significant alcohol consumption during the study, were taking steatogenic medications, possessed incomplete medical records or inadequate ultrasound images, or were lost to follow-up.

In this study, 946 patients with T2DM and MASLD were ultimately included. Hepatic elastography and LSM was performed at initial diagnosis and monitored during the follow-up period. All participants were advised on lifestyle modifications including hypocaloric diets and regular exercise, alongside their prescribed antidiabetic medications. Participants were categorized into progression and non-progression groups based on an increase in LSM value of more than 2 kPa observed at the three-year follow-up.

### 2.4 Ultrasound image acquisition

Ultrasound examinations were performed using an ACUSON Sequoia Ultrasound System (Siemens Healthineers, Erlangen, Germany). Patients underwent fasting for at least 3 h prior to their examinations to facilitate optimal imaging clarity. The procedure began with a conventional B-mode ultrasound to visualize liver structure, followed by a transition to shear wave elastography (SWE) for the assessment of liver stiffness. During SWE, breath-holding by patients was mandated to diminish measurement variability due to respiratory motion. Point shear wave elastography (pSWE) then quantified LSM values. For accuracy, SWE images targeting the right lobe of the liver, carefully avoiding blood vessels, were recorded. These images displayed a color-coded elastogram ranging from deep blue, indicating areas of lower stiffness, to red, denoting regions of higher stiffness, and were subsequently stored for detailed analysis.

### 2.5 Clinical data collection

A structured database was developed to collect LSM values along with a comprehensive range of clinical data. This included demographic and medical history information such as age, gender, history of hypertension, and smoking status. Anthropometric measurements, notably body mass index (BMI) and waist circumference (WC), were recorded at baseline and at follow-up. A broad spectrum of laboratory parameters at baseline was analyzed for detailed assessment, comprising hepatic function markers (aspartate aminotransferase [AST], alanine aminotransferase [ALT], albumin [ALB], platelet count [PLT]), indices of glucose metabolism (fasting blood glucose [FBG], glycosylated hemoglobin [HbA1c]), lipid profiles (total cholesterol [TC], high-density lipoprotein cholesterol [HDL-C], low-density lipoprotein cholesterol [LDL-C], triglycerides), and insulin resistance, assessed through the homeostasis model of insulin resistance (HOMA-IR). Additionally, medication use during the study period was documented to assess its impact on fibrosis progression.

### 2.6 Data preparation for DL model

**Fig. 1:**
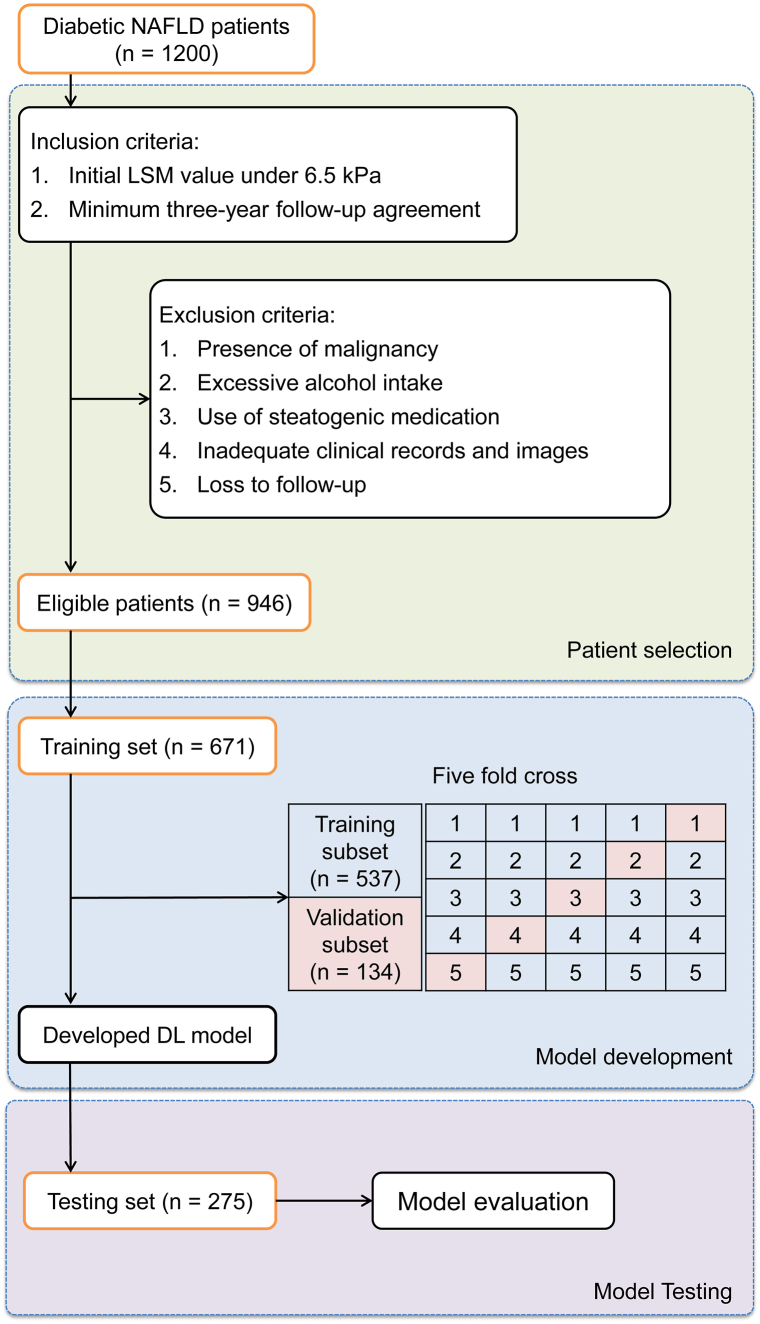
Workflow of patient selection, model development, and evaluation for diabetic MASLD patients.

Fig. 1 illustrates the workflow for patient selection and the subsequent development and evaluation phases of predictive models in this study. To ensure balanced training and testing datasets, we employed a stratified randomization method based on BMI and LSM. Patients were divided into three BMI categories: <25 kg/m^2^, 25–30 kg/m^2^, and >30 kg/m^2^, and three LSM categories: <4.5 kPa, 4.5–5.5 kPa, and >5.5 kPa. This resulted in nine distinct strata. Within each stratum, patients were randomly assigned to either the training set (70 %) or the testing set (30 %) using computer-generated random numbers. Clinical outcomes were determined based on their progression status; those in the progression group were assigned a value of “1″, and those in the non-progression group were assigned a value of “0". A stratified five-fold cross-validation method was applied to enhance model robustness by segmenting the training dataset into five equal portions, with each portion sequentially serving as a validation subset while the remainder functioned as the training subset. The performance of the model in each fold was assessed by selecting the iteration with the lowest validation loss for further analysis.

### 2.7 Pre-processing of hepatic SWE image

In preparation for neural network analysis, a total of 946 hepatic SWE images were collected from diabetic MASLD patients. Each segment was resized to 224 × 224 pixels via bilinear interpolation to enhance computational efficiency. The preprocessing of images included smoothing with Gaussian filters and normalization of pixel intensities to zero mean and unit variance to standardize input data for the neural network. For the training dataset, comprehensive data augmentation strategies were implemented to improve model generalization and reduce overfitting risks. These strategies included random flips, rotations ranging from −20 to 20°, and translations up to 15 % of the image size. Each training image underwent five variations for each of these three augmentation techniques, effectively increasing the size of the training set by 15 times to approximately 10,065 images.

### 2.8 DL model development

To address the challenge of predicting future fibrotic progression in diabetic MASLD patients, a DL integrated model (DI-model) was developed, incorporating both hepatic SWE images and clinical data. This model consists of two primary components. The first component (Image model) utilizes the pre-trained ResNet-50 architecture, renowned for its feature extraction capabilities, initially optimized on the extensive ImageNet dataset [17]. Modifications to this component included the introduction of a fully connected layer designed to reduce the feature dimensionality to 64, incorporating a rectified linear unit with a 0.2 dropout rate to prevent overfitting, and a sigmoid layer to predict the risk of fibrotic progression from hepatic SWE images. Optimization was performed using stochastic gradient descent (SGD) with a momentum of 0.9 and a binary cross-entropy loss. The initial learning rate was set at 0.001, adjusted in response to validation loss plateaus over ten epochs. Training included a protocol where each fold was permitted up to 100 epochs, with an early stopping mechanism activated after seven epochs without improvement in validation loss to prevent overfitting and enhance training efficiency. The optimal model was selected based on the lowest validation loss across all folds, thereby ensuring a balance between accuracy and computational efficiency.

In the second component (Tabular model) of the DI-model, the TabNet architecture was employed for sophisticated tabular data analysis. This architecture is adept at constructing predictive models utilizing extensive database information [18], aiding in the assessment of liver fibrotic progression risk from recorded clinical data. Optimization of the TabNet model involved adjustment of hyperparameters, including setting a learning rate of 0.1 and employing the Adagrad optimizer for adaptive adjustments. The training protocol specified a batch size of 16 and a virtual batch size of 8, with an early stopping mechanism activated after 50 epochs without improvement in validation loss, allowing each fold to be trained for up to 1000 epochs. The optimal model was determined based on the lowest loss observed across all folds on the validation set.

**Fig. 2:**
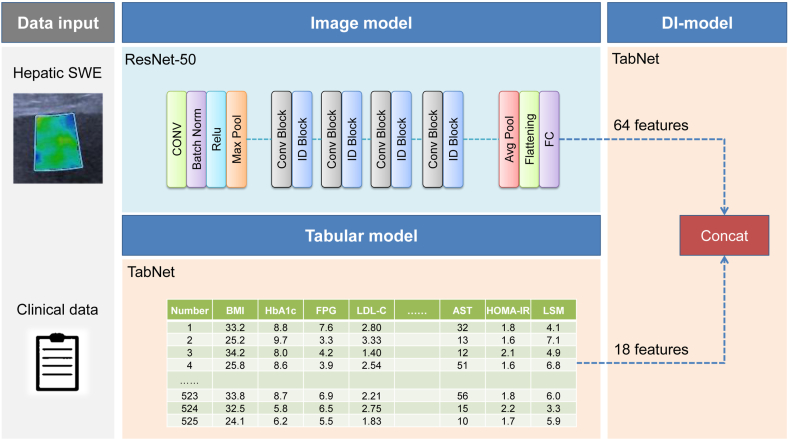
Architecture of the DI-model illustrating the dual-input streams with hepatic SWE images processed through ResNet-50 within the Image model and clinical plus LSM data processed through TabNet in the Tabular model. The final integration involves concatenating features from both streams before further refinement using the TabNet architecture.

The integration component of the DI-model concatenated the 64 features from the fully connected layer of the Image model with the clinical data processed by the Tabular model. Subsequently, the TabNet architecture was re-engaged with identical fine-tuning parameters for further refinement of the model. The best performing model, characterized by the lowest validation loss across all folds, was selected as the final implementation of the DI-model. The structural composition of the model is illustrated in Fig. 2.

### 2.9 Evaluation and comparative methods

Each DL model employed a sigmoid function to generate probabilistic scores reflecting the risk of fibrotic progression. Scores near 1 indicate higher risk, while scores close to 0 suggest lower risk. We used a threshold of 0.5 for delineation, as it is a widely accepted default value. This threshold is typically used when there is no strong reason to favor false positives over false negatives, simplifying both the model's training and its interpretation. Performance assessment and comparison were undertaken on the testing dataset, with the DI-model evaluated against (1) single-source data models (Image and Tabular models independently) and (2) a logistic regression model utilizing clinical data. Model efficacy was assessed by aligning predictions with actual outcomes in the testing set, focusing on discrimination, calibration, and clinical utility, which evaluate continuous outputs of the models across different threshold levels. Additionally, the performance of various models was compared using metrics derived from the confusion matrix to further elucidate their advantages and limitations. The selection of the optimal model was based on a comprehensive synthesis of these evaluations, with a priority given to models that exhibit superior discrimination, satisfactory calibration, and significant clinical utility. Finally, we employed the Shapley additive explanations (SHAP) approach to identify the top ten contributory factors in the best-performing model, thereby deepening our understanding of the key predictors of fibrotic progression.

### 2.10 Statistical analysis

The presentation of categorical variables is standardized as n (%), while continuous variables are reported as either mean ± standard deviation or median (interquartile range, IQR), depending on their distribution. Differences between the training and testing datasets were assessed using the Chi-square test for categorical variables, the independent-sample *t*-test for normally distributed continuous variables, and the Mann–Whitney *U* test for non-normally distributed continuous variables. The development of the logistic regression model was based on the univariate and multivariate logistic regression analyses that identified potential predictors of liver fibrotic progression and their odds ratios (OR). Model efficacy was assessed by analyzing the receiver operating characteristic (ROC) curve and the Precision-Recall (PR) curve, with the area under the curve (AUC) serving as a metric for discrimination ability. The DeLong test was used to statistically compare the AUCs of different models. Calibration of each model was evaluated through calibration curve analysis, complemented by the Brier Score (BS) and the Hosmer-Lemeshow (HL) test to assess model fit accuracy. Decision curve analysis (DCA) was employed to evaluate clinical benefits across different probability thresholds, establishing the clinical relevance of the models. Additionally, key performance metrics derived from the confusion matrix, including accuracy, precision, recall, kappa and the F1 score, were analyzed. These analyses were conducted using Python version 3.11.4, with a significance threshold set at a *p*-value of less than 0.05.

## 3 Result

### 3.1 Patient baseline characteristics

In the examined cohort of 946 diabetic MASLD patients, fibrotic progression was observed in 171 cases after three years, corresponding to an incidence rate of 18.1 %. To develop predictive models, 671 patients were allocated to the training dataset to train the models, and 275 were assigned to the testing dataset for model validation. Comparative analyses of the patients' demographic, clinical, and LSM data, and medication use, as well as the proportion of fibrotic progression, summarized in Supplementary Table S1, revealed no significant differences between the datasets (all *p*-values >0.05).

### 3.2 Predictors for fibrotic progression

In the training dataset, baseline predictive factors for fibrotic progression were assessed using logistic regression analyses. Elevated BMI, WC, LDL-C, FBG, HbA1c, HOMA-IR, and LSM, alongside reduced PLT, were significantly associated with fibrotic progression, as detailed in Table 1 (all *p*-values <0.05). Subsequent multivariate logistic regression, detailed in Table 2, identified independent predictors of fibrotic progression over three years. Notable predictors included increases in BMI, LDL-C, HbA1c, HOMA-IR, and LSM (all *p*-values <0.05). These variables served as independent predictors in a generalized linear logistic model for predicting fibrotic progression in diabetic MASLD patients.

**Table 1:** Univariate logistic regression analysis of characteristics between non-progression and progression groups in the training dataset.

Variable	Non-progression group (n = 558)	Progression group (n = 113)	Univariate logistic regression	
			*p*-value	OR
Demographic and medical history				
Age, years	45 (40, 51)	46 (39, 51)	0.137	1.018
Male gender, n(%)	322 (57.7 %)	57 (50.4 %)	0.156	0.746
Hypertension, n(%)	271 (48.6 %)	57 (50.4 %)	0.716	1.078
Smoking, n(%)	124 (22.2 %)	36 (31.9 %)	0.029	1.636
Anthropometric measurement				
Baseline BMI, kg/m^2^	29.36 ± 4.84	30.86 ± 5.65	0.004	1.060
Follow-up BMI	28.86 ± 4.84	30.40 ± 5.14	0.003	1.042
Baseline WC, cm	96.32 ± 15.02	99.89 ± 11.47	0.018	1.017
Follow-up WC, cm	95.04 ± 14.82	98.60 ± 11.52	0.021	1.012
Baseline laboratory parameters				
LDL-C, mmol/L	2.48 ± 0.84	2.65 ± 0.70	0.043	1.294
HDL-C, mmol/L	1.04 ± 0.39	1.02 ± 0.44	0.707	0.907
TC, mmol/L	3.97 ± 1.31	3.94 ± 1.47	0.820	0.983
Triglycerides, mmol/L	1.67 (1.24, 2.64)	1.74 (1.18, 2.56)	0.127	0.893
FBG, mmol/L	6.5 (4.9, 7.8)	7.2 (5.6, 9.2)	0.028	1.077
HbA1c, %	7.0 (5.8, 8.2)	7.2 (5.9, 9.4)	0.001	1.205
ALT, IU/L	31.5 (13.0, 49.0)	29.0 (9.0, 48.0)	0.250	0.995
AST, IU/L	19.5 (13.0, 39.0)	19.0 (14.0, 32.5)	0.346	0.997
PLT, × 10^9^/L	253 (221, 282)	244 (199, 273)	0.013	0.995
ALB, IU/L	40 (35, 45)	39 (35, 42)	0.500	0.990
HOMA-IR	1.8 (1.3, 2.2)	2.0 (1.6, 2.4)	0.006	1.587
Baseline LSM, kPa	4.6 (4.2, 5.0)	4.8 (4.4, 5.2)	0.001	1.792
Medication use, n (%)				
Metformin	519 (93.0 %)	107 (94.7 %)	0.516	1.340
Sulfonylurea	234 (41.9 %)	41 (36.3 %)	0.266	0.788
DPP4 inhibitor	84 (15.1 %)	23 (20.4 %)	0.162	1.442
Insulin	184 (33.0 %)	45 (39.8 %)	0.152	1.345
ACE inhibitor	262 (47.0 %)	59 (52.2 %)	0.308	1.234
ARB	133 (23.8 %)	33 (29.2 %)	0.229	1.318
Statin	317 (56.8 %)	72 (63.7 %)	0.176	1.335

**Table 2:** Multivariate logistic regression analysis identifying independent predictors of fibrotic progression in the training dataset.

Variables	*p*-value	OR	95 % CI
Baseline BMI	0.002	1.069	1.025–1.116
Baseline WC	0.163	1.011	0.996–1.026
Smoking	0.094	1.496	0.934–2.398
LDL-C	0.021	1.382	1.051–1.817
FBG	0.274	1.043	0.967–1.125
HbA1c	0.001	1.224	1.091–1.373
PLT	9.185	0.997	0.993–1.001
HOMA-IR	0.002	1.740	1.218–2.487
Baseline LSM	<0.001	1.970	1.365–2.843

### 3.3 Cross-validation of DL models

**Fig. 3:**
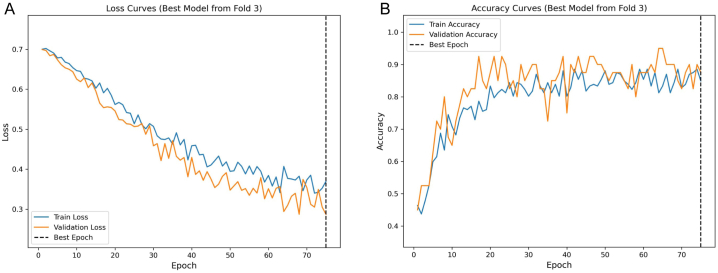
Validation dynamics of the optimal image model from the third fold of cross-validation. **Panel A** presents the convergence of training and validation loss, with a dashed line marking the epoch of minimum loss. **Panel B** shows the evolution of training and validation accuracy, with the dashed line highlighting the accuracy corresponding to the epoch of the lowest validation loss.

During the evaluation of the image model using cross-validation, the training process for each fold was comprehensively documented by recording the epoch-wise validation loss and validation accuracy. It was observed that during the initial epochs, both validation loss and accuracy demonstrated notable changes, which subsequently stabilized. An early stopping mechanism was set to activate after seven consecutive epochs without improvement, which led to the termination of training at epoch 69, 54, 75, 72, and 37 for folds one through five, respectively. The model from the third fold, which terminated training at epoch 75, recorded the lowest validation loss of 0.287, compared to the other folds which recorded losses of 0.378, 0.392, 0.335, and 0.557, respectively. Therefore, this fold was chosen to save the optimal image model. The cross-validation results, illustrated in Fig. 3A, show the convergence of training and validation loss. Fig. 3B presents the evolution of training and validation accuracy, corresponding to the epoch of the lowest validation loss, with a validation accuracy of 0.875.

**Fig. 4:**
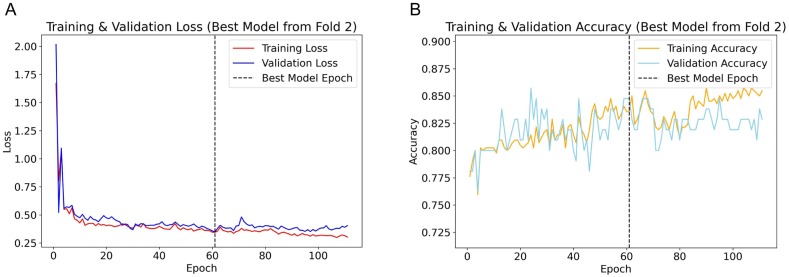
Cross-validation analysis for the optimal tabular model from fold 2. **Panel A** illustrates the trajectory of training and validation loss, delineating a marked decline until stabilization, with the dashed line indicating the epoch of the best-performing model. **Panel B** details the training and validation accuracy across epochs, with the dashed line marking the validation accuracy corresponding to the epoch with the lowest validation loss.

In the cross-validation of the Tabular model, a trend towards stabilization in the validation loss was similarly observed across all folds. Training cessation occurred at epochs 89, 110, 117, 119, and 71 for folds one through five, respectively. Notably, the model from the second fold outperformed others, documenting its lowest validation loss at epoch 60, registering at 0.349, lower than the minimal losses of 0.401, 0.356, 0.362, and 0.422 recorded in the other folds. Consequently, this model was selected as the optimal tabular model. Fig. 4A illustrates the loss trajectory, while Fig. 4B shows the accuracy trajectory, with the optimal model achieving a validation accuracy of 0.848.

**Fig. 5:**
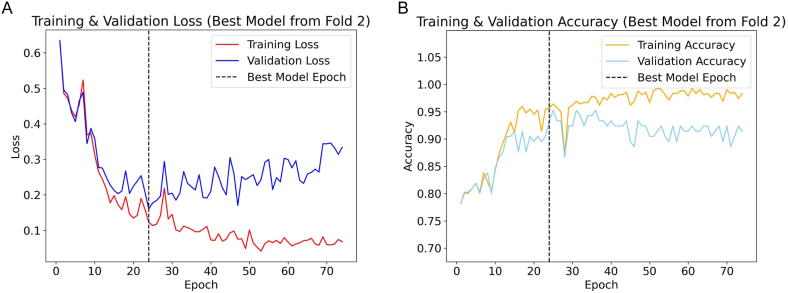
Cross-validation performance metrics for the DI-Model in fold 2. **Panel A** conveys the training and validation loss, illustrating a descent to stability, marked by a dashed line at the epoch with optimal performance. **Panel B** maps the training and validation accuracy, with a dashed line marking the epoch corresponding to the lowest validation loss and its associated validation accuracy.

The DI-model, which integrated features from the fully connected layer of the Image model with clinical data, demonstrated superior performance in cross-validation. The model reached its peak performance in the second fold, recording a validation loss of 0.161 and an accuracy of 0.933, as shown in Fig. 5A and B, respectively. The lowest validation losses in the other folds were 0.174, 0.168, 0.178, and 0.175, respectively. These results consistently exceeded those of the individual Image and Tabular models, indicating a higher predictive accuracy of the integrated model across diverse datasets.

### 3.4 Model evaluation via testing dataset

**Fig. 6:**
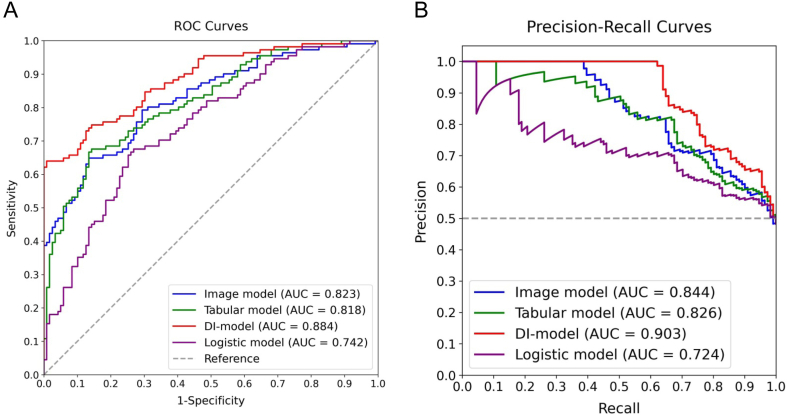
Comparative performance analysis of Image, Tabular, DI, and Logistic models using the testing dataset: **A)** ROC curves with AUC values, **B)** PR curves with AUC values.

**Fig. 7:**
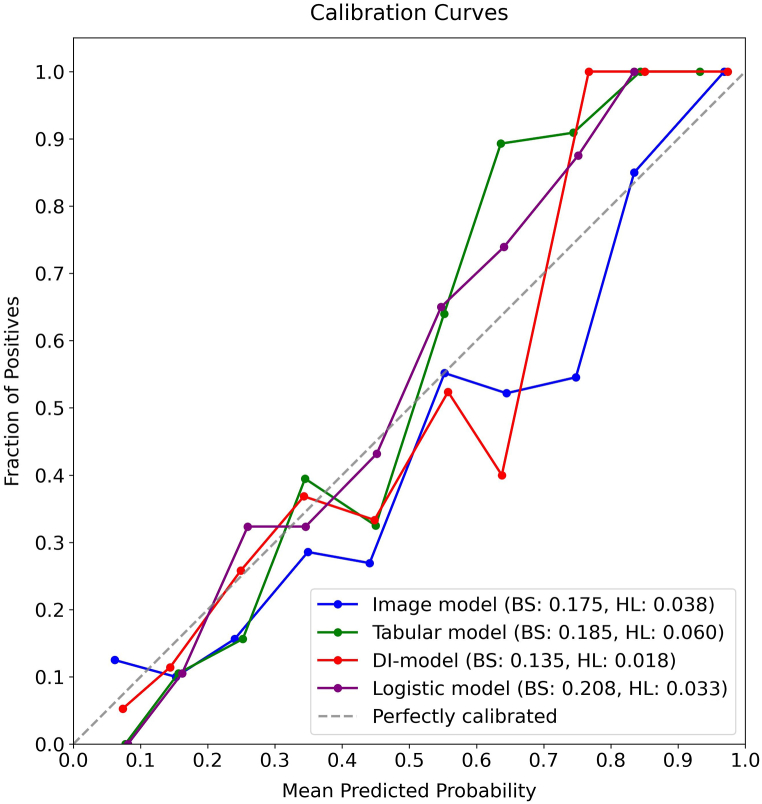
Calibration curves for Image, Tabular, DI, and Logistic models using the testing dataset. The curves illustrate the agreement between predicted probabilities and observed outcomes. The BS and HL statistics are provided for each model to indicate the goodness-of-fit of the probabilistic predictions. Lower BS and HL values indicate better calibration and model performance.

**Fig. 8:**
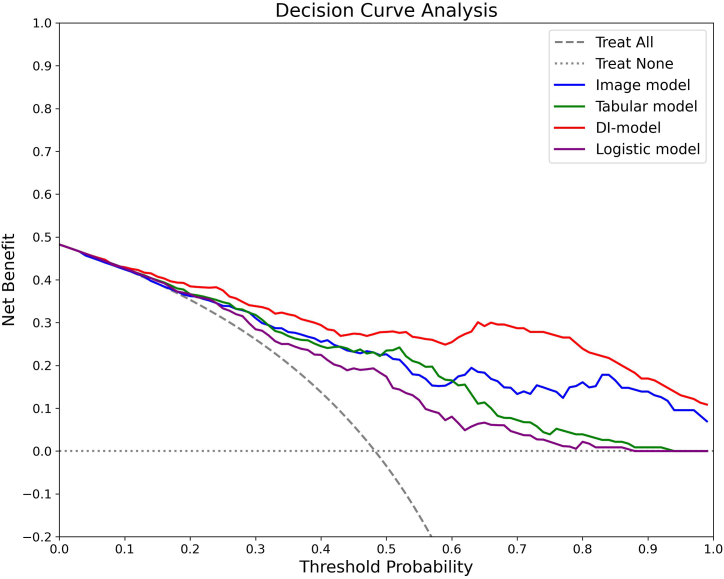
DCA for Image, Tabular, DI, and Logistic models using the testing dataset. The analysis assesses the net benefit of each model across a range of threshold probabilities, comparing the clinical usefulness of each model's predictions.

**Fig. 9:**
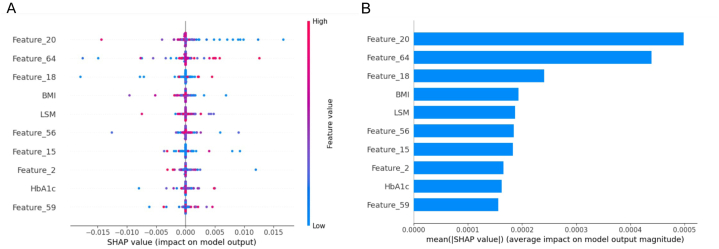
Shapley analysis of the top 10 predictive features in the DI-model. **A)** Bee swarm plot representing the distribution of SHAP values for each feature and their respective impact on the model's output, and **B)** Bar chart depicting the mean absolute SHAP values, quantifying the average influence of these features on the predictive accuracy of the model.

In the testing dataset, a detailed evaluation was conducted comparing the Image model, Tabular model, DI-model, and Logistic model, focusing on discrimination, calibration, and clinical utility. Additionally, a comparative analysis based on the confusion matrix was summarized in Table 3. As shown in Fig. 6A and B, the DI-model demonstrated superior discrimination, achieving AUCs of 0.884 and 0.903 for ROC and PR curves, respectively, which were significantly higher than those of the other models (all *p*-values <0.05). As illustrated in Fig. 7, its calibration curve closely followed the reference line, with the lowest BS of 0.135 and HL values of 0.018. Fig. 8 shows that the largest area under the DCA curve reflected its optimal clinical utility. These findings collectively demonstrate that the DI-model outperformed the Image, Tabular, and Logistic models, which exhibited minor differences in their respective efficacies. Additionally, superior accuracy, F1-score, and kappa value further highlighted the robustness of the DI-model. A shapley analysis, illustrated by a bee swarm plot in Fig. 9A and a bar chart in Fig. 9B, identified the top ten contributors to the predictive accuracy of the DI-model. The analysis highlighted that in addition to seven image features derived from hepatic SWE, clinical predictors such as BMI, LSM, and HbA1c were critical in predicting the progression of liver fibrosis.

**Table 3:** Predictive performance of Image, Tabular, DI, and Logistic models on the testing dataset.

Model	Accuracy (95 % CI)	Precision (95 % CI)	Recall (95 % CI)	Kappa (95 % CI)	F1 Score (95 % CI)
Image model	0.743 (0.683, 0.800)	0.706 (0.629, 0.782)	0.802 (0.726, 0.873)	0.589 (0.478, 0.700)	0.751 (0.689, 0.807)
Tabular model	0.752 (0.700, 0.809)	0.776 (0.689, 0.854)	0.685 (0.593, 0.770)	0.602 (0.481, 0.703)	0.727 (0.653, 0.788)
DI model	0.796 (0.743, 0.848)	0.814 (0.728, 0.883)	0.748 (0.667, 0.822)	0.690 (0.575, 0.787)	0.779 (0.709, 0.837)
Logistic model	0.691 (0.630, 0.752)	0.708 (0.615, 0.796)	0.613 (0.524, 0.705)	0.479 (0.384, 0.607)	0.657 (0.579, 0.726)

## 4 Discussion

In this study, we sought to accurately predict the risk of fibrotic progression among diabetic MASLD patients initially presenting no hepatic fibrosis. Given the severe consequences associated with advanced fibrosis, such as decompensated cirrhosis and portal hypertension [19,20], identifying at-risk individuals at an earlier stage is critical for timely intervention. Our approach involved the use of DL frameworks to analyze both clinical data and hepatic SWE images. An integrated model, named the DI-model, was developed and demonstrated superior performance in predicting fibrotic progression by leveraging combined data sources. This model outperformed other predictive models constructed with either data type alone. The innovation of our study lies in its demonstration that DL technology can significantly refine the predictive accuracy of future fibrotic progression based on routine clinical evaluations. This enhanced predictive capability is crucial for optimizing treatment strategies earlier in the disease course, potentially preventing severe liver damage and improving patient outcomes.

The progression of MASLD is highlighted by its association with increased mortality from liver-related conditions and is exacerbated by T2DM [21]. Recent meta-analyses from paired-biopsy studies indicate that up to 30 % of MASLD patients progress at least one stage of fibrosis over a decade, demonstrating that steatosis does not remain static [22]. Comorbid T2DM exacerbates this progression, as it introduces additional risk factors such as insulin resistance and dyslipidemia, which in turn promote fatty acid accumulation and enhance inflammatory responses [23,24]. These processes collectively advance hepatic steatosis, propelling the progression of MASLD towards more severe fibrotic stages. Consequently, the risk of fibrosis progression in diabetic MASLD patients over three years particularly warrants increased attention. The choice of a three-year follow-up period in our study aims to balance the need for a sufficient timeframe to observe meaningful changes in liver fibrosis with the practical considerations of patient retention and compliance in a longitudinal study. A three-year period helps avoid the issues of patient attrition and incomplete data that longer follow-ups might cause while allowing for the detection of significant disease progression in a notable proportion of patients. The previous study by Wong et al. [25] revealed, using paired liver biopsies, that 27 % of MASLD patients with simple steatosis had fibrosis progression over three years. Furthermore, the current guidelines of the American Gastroenterological Association recommend monitoring MASLD every three years using non-invasive tests [26]. These guidelines are supported by extensive histological studies and expert consensus, indicating that a three-year interval is both clinically relevant and practical for assessing fibrosis progression.

In this study, LSM was employed over liver biopsy for monitoring hepatic fibrosis, given its widespread recognition as a non-invasive diagnostic tool for this condition [27]. The threshold of LSM set below 6.5 kPa to exclude advanced fibrosis is endorsed by the research from Siddiqui et al. [11], which confirmed that this cut-off effectively excludes advanced fibrosis with a negative predictive value of 0.91, based on liver histology as the gold standard. An increase in LSM value > 2 kPa was defined as fibrosis progression because, in patients with an initial LSM under 6.5 kPa, such an increase corresponds to a 30 % elevation, suggesting significant changes in liver stiffness rather than mere measurement variability. Furthermore, Lee et al. [28] reported that among diabetic MASLD patients without advanced fibrosis at baseline, 85.7 % of those who progressed to advanced fibrosis over three years experienced an LSM increase of >2 kPa. This target population closely resembles ours, focusing on diabetic MASLD patients with simple steatosis and their fibrosis progression. Within this framework, our study revealed that 18.1 % of diabetic MASLD patients without initial signs of advanced hepatic fibrosis exhibited progression over three years. This incidence is comparable to Wong et al. [25] who demonstrated through paired liver biopsies over three years that simple steatosis is not always quiescent. In their study, 23 % of patients with simple steatosis developed NASH, and 28 % had fibrosis progression after 3 years. Similarly, the findings are in line with the latest report by Xia et al. [29], who observed a progression rate of nearly 15 % using LSM as a diagnostic marker for fibrosis progression. Such a substantial rate of fibrosis progression highlights the critical need for early prediction and intervention in this patient cohort.

Cross-sectional studies frequently utilize non-invasive methods for classifying liver fibrosis, yet longitudinal research on fibrosis progression in MASLD patients initially without fibrosis remains limited. Recently developed scoring systems utilizing clinical and serological markers generally predict disease progression in NASH effectively [[30], [31], [32]], but their efficacy is reduced in mild to moderate MASLD stages [33]. In contrast, liver elastography has shown superior predictive ability over these systems. For instance, research by Ajmera et al. [34] indicated that a 15 % increase in liver stiffness measured by magnetic resonance elastography suggests a shift from early to advanced fibrosis. With advancements in artificial intelligence, new models have improved the extraction of predictive insights from clinical data. Ghandian et al. [35] demonstrated the capability of machine learning algorithms to identify early-stage MASLD patients at high risk of progressing to NASH, using routine clinical data. Our study employs DL technology, integrating baseline hepatic SWE images with clinically significant predictors, to predict fibrosis progression over three years in diabetic MASLD patients initially free from advanced fibrosis. This methodology does not increase the clinical examination burden and facilitates the implementation of a predictive model suitable for broad clinical application.

Our findings illustrate that the DI-model, which integrates both clinical and imaging data, exhibits enhanced predictive capabilities for fibrotic progression compared to models utilizing singular data types. This superiority likely arises from the complex and nuanced nature of fibrotic changes at intermediate stages, which may not be adequately captured by either clinical parameters or SWE images alone. The integration of diverse data sources in the DI-model facilitates a more comprehensive analysis of these subtle changes. Moreover, the architecture of the DI-model, particularly the feature fusion module, is tailored to balance the disparate feature dimensions by reducing the feature count from the original 1000 in the image model's full connection layer to 64. This design choice prevents the dilution of contributions from the smaller feature set. An in-depth Shapley value analysis of the top ten features influencing the DI-model reveals the significance of clinical indicators such as LSM, BMI, and HbA1c. These factors highlight the critical role of metabolic control and body weight management in the progression of liver fibrosis, suggesting that these parameters exacerbate the progression of MASLD.

The developed DI-model has shown promising results in discrimination, calibration, and clinical applicability. Specifically, the DI-model provides a percentage risk prediction for each patient regarding the likelihood of fibrosis progression over three years. High-risk patients identified by the model can receive more intensive monitoring and interventions, such as stricter diet, exercise plans, and potentially pharmacological treatment. This percentage risk output aids in personalized patient counseling and helps track changes in fibrosis risk over time. By dynamically recording the patient's fibrosis risk percentage, clinicians can adjust intervention plans based on whether the risk increases or decreases, optimizing clinical outcomes and resource allocation. Furthermore, the model helps streamline resource allocation in healthcare settings by effectively identifying high-risk individuals for targeted interventions. Given the large MASLD patient population, this approach ensures that efforts are focused on those most likely to benefit from intensive management, ultimately enhancing patient outcomes and healthcare efficiency.

Nonetheless, there are notable limitations that should be addressed. Firstly, the single-center design and the relatively small patient cohort, resulting from strict inclusion and exclusion criteria, may restrict the broader applicability of the findings. Additionally, the elastography images and LSM values obtained using our device have not been compared with other devices, which may further limit the generalizability of our results. Secondly, while LSM is widely accepted for the clinical diagnosis of liver fibrosis and shows comparable diagnostic accuracy to liver biopsy [36,37], the absence of biopsy confirmation in this study introduces some diagnostic uncertainty. Thirdly, CNNs inherently present a “black box” problem, where the features extracted during training are often represented as numerical identifiers. These features correspond to complex patterns learned from the data, rather than easily describable clinical concepts, making direct clinical interpretation challenging. Finally, although all patients were recommended to adhere to a hypocaloric diet and exercise regimen, the variability in individual adherence and the lack of personalized, quantifiable diet and exercise plans based on each patient's specific conditions could influence clinical outcomes. This variability might introduce bias and affect the accuracy of the model. Despite our confidence in the DI-model's ability to identify patients at risk of fibrosis progression, future research should focus on conducting larger, multicenter studies with external validation to improve the robustness and generalizability of the model.

In conclusion, the study has successfully developed a DL-based neural network, termed the DI-model, to integrate hepatic SWE images and clinical data for predicting fibrotic progression risk in diabetic MASLD patients without initial hepatic fibrosis. This innovative approach, combining imaging and tabular data within a DL model, holds promise for more accurate quantification of fibrosis progression risk. Ongoing refinements to the DI-model are expected to further enhance its predictive capabilities based on routine clinical evaluations, enabling early adjustment of medical interventions and improved adherence to lifestyle interventions prior to advanced fibrosis development.

## Ethics and consent

This study adhered to the principles outlined in the Declaration of Helsinki and obtained approval from the medical ethics committee of Meng Cheng County Hospital of Chinese Medicine (MZY-XJS-201817). All patients provided written informed consent, which included permission for the publication of any images and clinical data in the manuscript.

## Funding sources

Not applicable.

## Data availability statement

All data generated or analyzed during this study are included in this article. Further enquiries can be directed to the corresponding author.

## CRediT authorship contribution statement

**Ruihong Dai:** Writing – review & editing, Writing – original draft, Visualization, Supervision, Conceptualization. **Miaomiao Sun:** Writing – original draft, Software, Methodology, Formal analysis, Data curation, Conceptualization. **Mei Lu:** Visualization, Validation, Software, Resources, Methodology, Formal analysis. **Lanhua Deng:** Validation, Software, Methodology, Formal analysis, Data curation, Conceptualization.

## Declaration of competing interest

The authors declare that they have no known competing financial interests or personal relationships that could have appeared to influence the work reported in this paper.
