# Supplementary material for: Deep learning for predicting fibrotic progression risk in diabetic individuals with metabolic dysfunction-associated steatotic liver disease initially free of hepatic fibrosis
Source: Heliyon. 2024 Jul 5;10(13):e34150. doi: 10.1016/j.heliyon.2024.e34150 (PMC11282990; doi:10.1016/j.heliyon.2024.e34150)
Supplement: Multimedia component 1 [file mmc1.docx]

**Supplementary**

**Table S1**. Comparison of baseline characteristics between training and testing datasets in diabetic NAFLD patients

| Variable | Training set (n = 671) | Testing set (n = 275) | *p-*value |
| --- | --- | --- | --- |
| Demographic and medical history |  |  |  |
| Age, years | 45 (39, 51) | 46 (40, 50) | 0.899^a^ |
| Male gender, n(%) | 379 (56.5%) | 161 (58.5%) | 0.561^b^ |
| Hypertension, n(%) | 328 (48.9%) | 127 (46.2%) | 0.450^b^ |
| Smoking, n(%) | 160 (23.8%) | 76 (27.6%) | 0.221^b^ |
| Anthropometric measurement |  |  |  |
| Baseline BMI, kg/m^2^ | 29.3 (26.1, 32.6) | 29.0 (26.0, 32.3) | 0.161^a^ |
| Follow-up BMI | 28.9 (25.6, 32.3) | 28.4 (25.2, 31.6) | 0.129 |
| Baseline WC, cm | 96.92±14.54 | 96.96±14.71 | 0.979^c^ |
| Follow-up WC, cm | 95.64±14.24 | 95.63±13.63 | 0.986 |
| Baseline laboratory parameters |  |  |  |
| LDL-C, mmol/L | 2.48±0.84 | 2.55±0.70 | 0.142^c^ |
| HDL-C, mmol/L | 1.04±0.38 | 1.02±0.44 | 0.708^c^ |
| TC, mmol/L | 3.97±1.31 | 3.94±1.46 | 0.820^c^ |
| Triglycerides, mmol/L | 1.68 (1.23, 2.62) | 1.78 (1.25, 2.63) | 0.611^a^ |
| FBG, mmol/L | 5.2 (3.7, 7.3) | 5.2 (3.7, 7.2) | 0.989^a^ |
| HbA1c, % | 7.0 (5.8, 8.3) | 6.9 (5.5, 8.4) | 0.427^a^ |
| ALT, IU/L | 40 (29, 55) | 37 (24, 70) | 0.435^a^ |
| AST, IU/L | 19 (12, 36) | 19 (13, 38) | 0.863^a^ |
| PLT, ×10^9^/L | 252 (217, 281) | 253 (216, 282) | 0.944 ^a^ |
| ALB, IU/L | 40 (35, 44) | 39 (34, 44) | 0.302^a^ |
| HOMA-IR | 1.8 (1.4, 2.3) | 1.8 (1.5, 2.3) | 0.349^a^ |
| Baseline LSM, kPa | 4.6 (4.2, 5.0) | 4.6 (4.2, 5.0) | 0.585^a^ |
| Medication use, n (%) |  |  |  |
| Metformin | 626 (93.3%) | 263 (95.6%) | 0.169 |
| Sulfonylurea | 275 (41.0%) | 119 (43.3%) | 0.517 |
| DPP4 inhibitor | 107 (15.9%) | 45 (16.4%) | 0.874 |
| Insulin | 229 (34.1%) | 104 (37.8%) | 0.281 |
| ACE inhibitor | 321 (47.8%) | 123 (44.7%) | 0.384 |
| ARB | 166 (24.7%) | 64 (23.3%) | 0.633 |
| Statin | 389 (58.0%) | 158 (57.5%) | 0.883 |
| Fibrotic progression, n(%) | 113 (16.7%) | 58 (21.1%) | 0.123^b^ |

^a^ for Mann–Whitney U test, ^b^ for Chi-square test, ^c^ for independent-sample t-test
